# Nitidine chloride, a benzophenanthridine alkaloid from *Zanthoxylum nitidum* (Roxb.) DC., exerts multiple beneficial properties, especially in tumors and inflammation-related diseases

**DOI:** 10.3389/fphar.2022.1046402

**Published:** 2022-11-24

**Authors:** Qiang Lu, Shuang Luo, Zhongfeng Shi, Mingzhen Yu, Weifeng Guo, Cailan Li

**Affiliations:** ^1^ Department of Pharmaceutical Sciences, Zunyi Medical University, Zhuhai Campus, Zhuhai, China; ^2^ Shenzhen Traditional Chinese Medicine Hospital, The Fourth Clinical Medical College of Guangzhou University of Chinese Medicine, Shenzhen, China; ^3^ New Drug Reserach and Development Center, Guangdong Pharmaceutical University, Guangzhou, China; ^4^ Department of Pharmacology, Zunyi Medical University, Zhuhai Campus, Zhuhai, China; ^5^ Key Laboratory of Basic Pharmacology of Ministry of Education and Joint International Research Laboratory of Ethnomedicine of Ministry of Education, Zunyi Medical University, Zunyi, China; ^6^ Key Laboratory of Basic Pharmacology of Guizhou Province and School of Pharmacy, Zunyi Medical University, Zunyi, China

**Keywords:** nitidine chloride, pharmacology, pharmacokinetics, toxicology, extraction, formulation

## Abstract

Plant-derived alkaloids are a kind of very important natural organic compounds. Nitidine chloride is one of the main active ingredients in *Zanthoxylum nitidum* (Roxb.) DC. which is a frequently-used Chinese herbal medicine. *Z. nitidum* has many kinds of efficacy, such as activating blood circulation and removing stasis, promoting qi circulation and relieving pain, and detoxication and detumescence. In China, *Z. nitidum* is usually used for the treatment of gastrointestinal diseases, toothache, and traumatic injury. At present, there are numerous studies of nitidine chloride with regard to its pharmacology, pharmacokinetics, toxicology, etc. However, a systematic, cutting-edge review of nitidine-related studies is extremely lacking. The present paper aimed at comprehensively summarizing the information on the extraction, separation and purification, pharmacology, pharmacokinetics, toxicology and formulation of nitidine chloride. The knowledge included in the present study were searched from the following academic databases involving Web of Science, PubMed, Google scholar, Elsevier, CNKI and Wanfang Data, till July 2022. In terms of nitidine chloride extraction, enzymatic method and ultrasonic method are recommended. Resin adsorption and chromatography were usually used for the separation and purification of nitidine chloride. Nitidine chloride possesses diversified therapeutical effects, such as anti-tumor, anti-inflammation, anti-colitis, anti-malaria, anti-osteoporosis, anti-rheumatoid and so on. According to pharmacokinetics, the intestinal absorption of nitidine chloride is passive diffusion, and it is rarely excreted with urine and feces in the form of prototype drug. Nitidine chloride has a moderate binding to plasma protein, which is independent of the drug concentration. As to toxicology, nitidine chloride showed certain toxicity on liver, kidney and heart. Certain new formulations, such as nanoparticle, microsphere and nano-micelle, could increase the therapeutic effect and decrease the toxicity of nitidine chloride. Despite limitations such as poor solubility, low bioavailability and certain toxicity, nitidine chloride is still a promising natural alkaloid for drug candidates. Extensive and intensive exploration on nitidine chloride is essential to promote the usage of nitidine-based drugs in the clinic practice.

## Introduction

Natural products mainly come from plants, animals and microorganisms, including phenylpropanoids, quinones, flavonoids, terpenoids, steroids, alkaloids, polysaccharides and so on (Atanasov et al., 2021). Among them, alkaloids are a kind of very important natural organic compounds exhibiting huge medicinal potential. With the discovery of the first alkaloid morphine in 1806, there has been about 130,000 known alkaloids (Efferth and Oesch, 2021). Studies have shown that alkaloids have a wide range of pharmacological effects, such as anti-inflammation, antioxidation, anti-cancer, immune regulation, and liver protection (Elsheep et al., 2022). There are more than 100 kinds of alkaloid drugs in clinical application, such as berberine, ephedrine, hyoscyamine, scopolamine, anisodamine, cocaine, and quinine (Li et al., 2022). At present, the research of alkaloids has always been one of the important research fields of pharmacy, natural products chemistry, medicinal chemistry, drug discovery, which has continuously attracted scientists’ interest and lasted for a long time.

**FIGURE 1:**
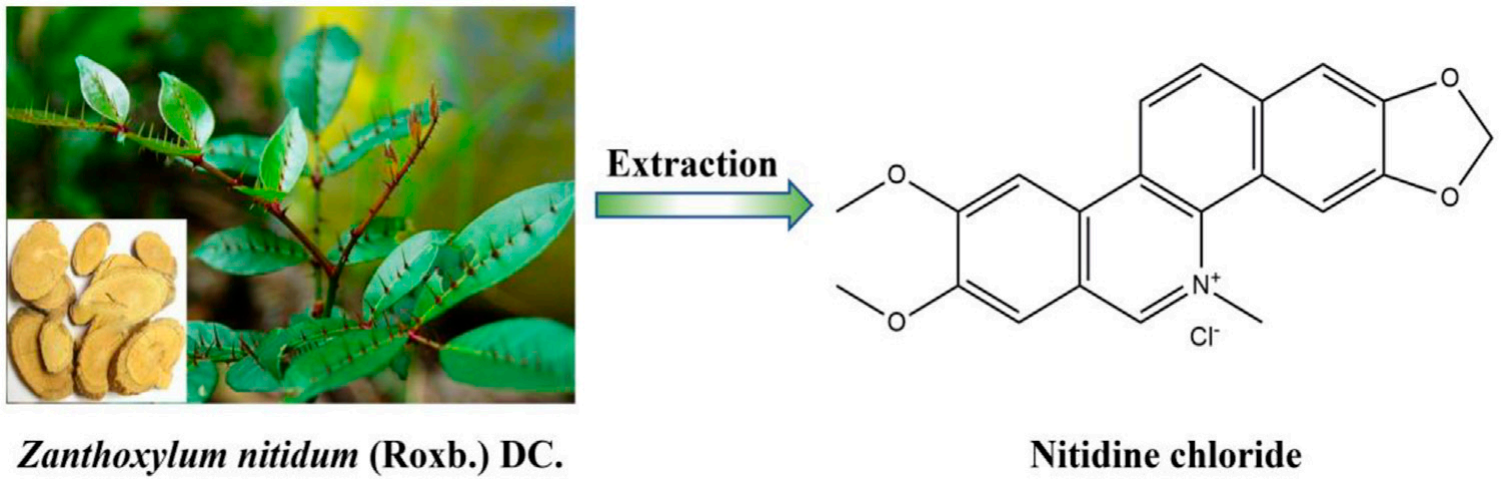
Origin plant and chemical structure of nitidine chloride.

Nitidine chloride (C_21_H_18_NO_4_·Cl, MW: 383.82, Figure 1), a quaternary ammonium benzophenanthridine alkaloid, isolated for the first time by Arthur et al., in 1959 from the roots of *Zanthoxylum nitidum* (Roxb.) DC., and then also found in many other medicinal plants, such as *Zanthoxylum dissitum* Hemsl., *Zanthoxylum myriacanthum* Wall. ex Hook. f., *Toddalia asiatica* (L.) Lam., *Broussonetia papyrifera* (Linnaeus) L'Heritier ex Ventenat, and *Phyllanthus muellerianus* (Kuntze) Excell (Cesari et al., 2015). At present, due to its relatively high content in *Z. nitidum*, nitidine chloride is mainly extracted from the roots or stems of *Z. nitidum* which is a common traditional Chinese medicine. *Z. nitidum* has many kinds of traditional efficacy, such as activating blood circulation and removing stasis, promoting qi circulation and relieving pain, expelling wind and dredging meridian, and detoxication and detumescence. In China, *Z. nitidum* is usually used for the treatment of gastrointestinal diseases, toothache, and traumatic injury (Lu et al., 2020a; Lu et al., 2020b).

Since its discovery, nitidine chloride has tremendously drawed the attention of many investigators. As a result, various studies on nitidine chloride have emerged one after another. Strikingly, nitidine chloride showed a wide range of pharmacological activities including anti-tumor, anti-inflammation, anti-colitis, anti-malaria, anti-osteoporosis and so on (Cui et al., 2020). However, a systematic, cutting-edge review of nitidine chloride studies is extremely lacking. Therefore, this paper makes a comprehensive and in-depth summary on the extraction, separation and purification, pharmacology, pharmacokinetics, toxicology and formulation of nitidine chloride, in order to provide a beneficial reference for the future research on nitidine chloride.

## Extraction of nitidine chloride

Nitidine chloride is a common benzophenanthridine alkaloid. At present, the extraction methods of nitidine chloride mainly include reflux method, enzymatic method and ultrasonic method.

### Reflux method

Among the extraction methods of nitidine chloride, reflux method is the most widely used, mainly including alcohol reflux and acid-alcohol reflux extraction. Lei et al. (2011) used 80% ethanol for reflux extraction and optimized the optimal extraction process by orthogonal design. Huang H. L et al. (2011) compared the extraction effects of alcohol reflux and acid-alcohol reflux, and the results showed that acid-alcohol reflux can greatly increase the extraction rate compared with ordinary alcohol reflux, which can promote the improvement of the extraction process of nitidine chloride. Zhao et al. (2015) extracted with 80% ethanol, 8 times the amount of medicinal herb, and added hydrochloric acid to adjust the pH to 5. The average extraction rate of total alkaloids was 12.8%, and the content of nitidine chloride reached 41 mg g^−1^. Acid-alcohol reflux can improve the extraction rate of nitidine chloride, but the long extraction time aggravates the acid corrosion of the equipment. Zeng et al. (2013, 2014) improved the acid-alcohol reflux process by adopting the internal boiling method, which not only reduced the extraction temperature and time, but also decreased the amount of acid and alcohol, relieving the corrosion to the equipment.

### Enzymatic method

At present, most alkaloids are extracted from plants. Because cellulase can first destroy the cell wall of plants, enzymatic method is an effective means to extract active ingredients (Sun et al., 2013). Lu and Li (2013a) first hydrolyzed *Z. nitidum* with mixed cellulase/pectinase, and then extracted it by acid-alcohol reflux at room temperature. In this study, the extraction yield of nitidine chloride was 85.96%. The enzyme pretreatment method can reduce the extraction times and the amount of solvent, and meet the production requirements of energy saving and environmental protection.

### Ultrasonic method

Ultrasonic extraction uses the cavitation, thermal effect and vibration produced by ultrasound to break the plant cell wall, accelerate the entry of organic solvents into the cell, and promote the dissolution of alkaloids into the solvent to achieve the purpose of extraction (Shirsath et al., 2012). At present, ultrasonic extraction is mainly used as an auxiliary method in extracting natural products. Lu and Li (2013b) used enzyme pretreatment and ultrasonic extraction to increase the extraction rate of nitidine chloride to 90.26%. The yield of dry extract can be significantly improved by ultrasound-enzyme-assisted semi-bionic extraction, which is suitable for mass production (Lu et al., 2014). Li et al. (2015) used the semi-bionic ultrasonic method for extraction. First, the compound enzyme was adopted for pretreatment, and then 60% ethanol containing citric acid and triethylamine was employed for ultrasonic extraction. The yield of nitidine chloride was 0.252%. This method can overcome the problem of poor water solubility of pure water buffer and promote the extraction rate.

The acid-alcohol reflux operation is simple and the extraction rate is high, but the corrosion of the equipment can not be ignored, which is unfavorable to large-scale industrial production. Enzymatic and ultrasonic methods not only keep the extraction rate, but also discard the use of acid, greatly reduce the use of solvent, and is more environmentally friendly while solving the corrosion problem.

## Separation and purification of nitidine chloride

There are many kinds of alkaloids in *Z. nitidum*, such as chelerythrine, skimmianine, magnoflorine, sanguinarine, nitidine chloride, oxynitidine, *etc.* In order to obtain purer nitidine chloride, it is necessary to further separate and purify it on the basis of extracting the crude product.

### Crystallization

Huang and Li (1980) and Wang (1981) treated the methanol extract of *Z. nitidum* and crystallized it statically to purify and precipitate nitidine chloride crystals. However, chloroform was used during the experiment, which caused great environmental pollution.

### Resin adsorption

Lu et al. (2010b) used Ls006 cation resin to enrich and purify nitidine chloride through static adsorption and desorption, with a purity of more than 90%. Zeng et al. (2014) extracted nitidine chloride from *Z. nitidum* by the adding-acid internal ebullition method, and then statically adsorbed it with 732 cationic resin and ultrasonic-assisted desorption. The purity of separated nitidine chloride was 94.5%. The resin purification method has outstanding effect, simple operation, low cost and high industrial application value.

### Chromatography

Huang et al. (2013) repeatedly ground the extracts of *Z. nitidum* roots with chloroform, and the resulting precipitate was dissolved in methanol and recrystallized. The crude product obtained by recrystallization is separated and purified by high performance liquid chromatography after secondary recrystallization, and eluted by C-18 chromatographic column to obtain nitidine chloride with a purity of more than 98%. The sample can be used as a chemical reference for quality control and scientific research.

Therefore, the methods of crystallization and resin adsorption are more suitable for industrial production of nitidine chloride, and the chromatography method is better applicable for laboratorial preparation and can achieve higher purity of nitidine chloride (Lu, 2010a; Liu and Qiu, 2019).

## Pharmacology of nitidine chloride

**FIGURE 2:**
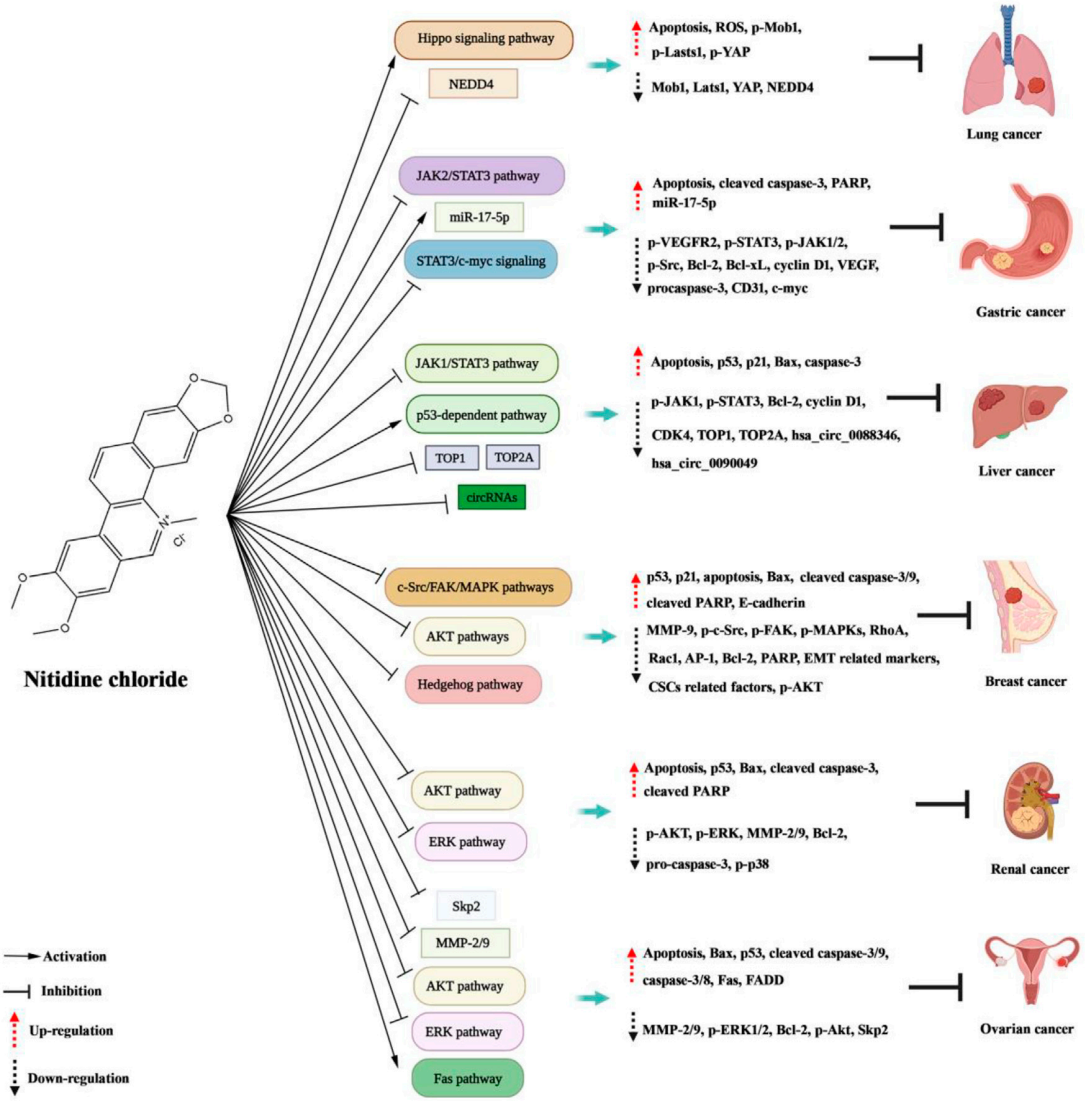
Molecular mechanisms of nitidine chloride against cancer.

**FIGURE 3:**
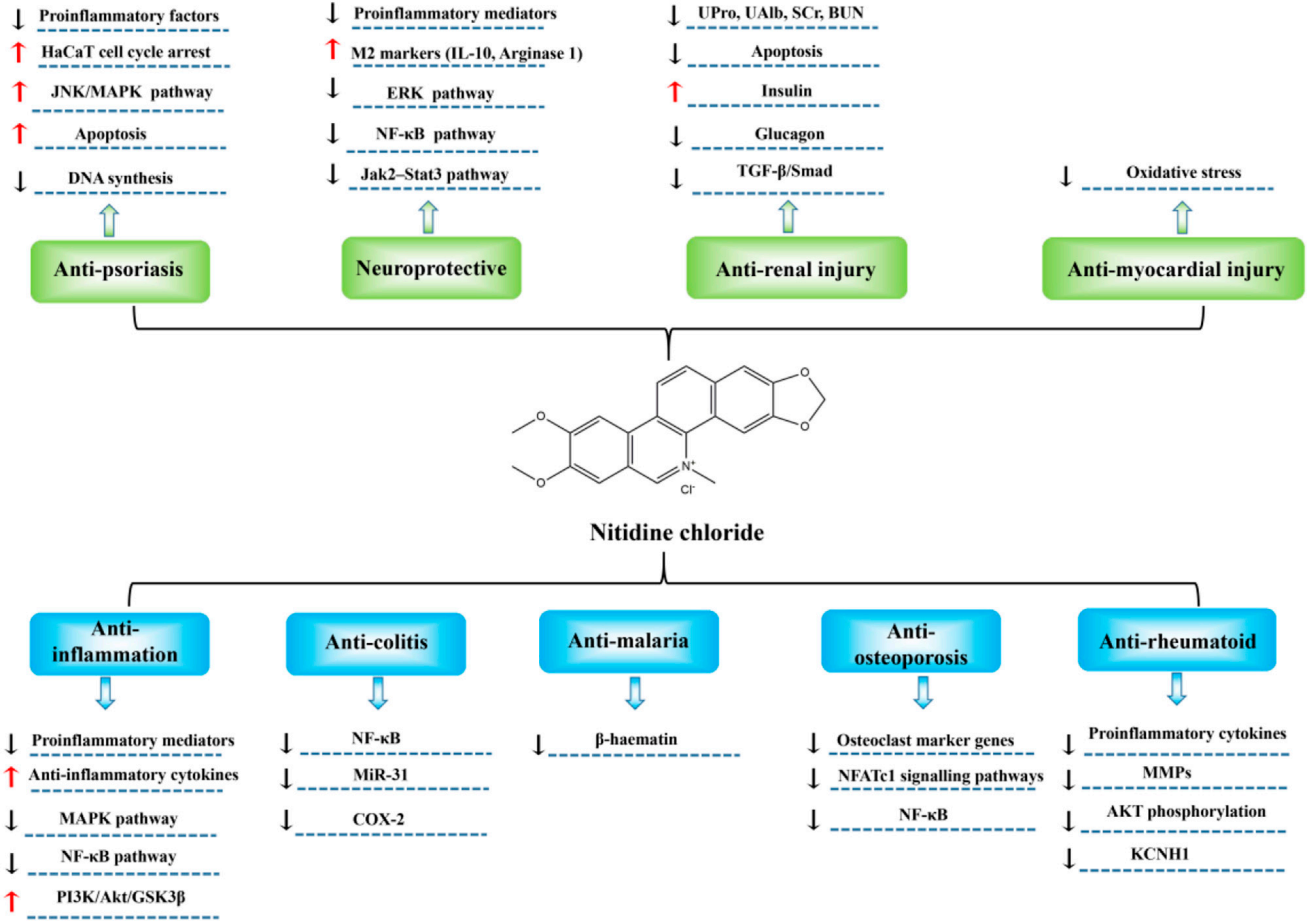
Molecular mechanisms of nitidine chloride in the treatment of non-cancer diseases.

Nitidine chloride shows excellent biological activity, and has significant inhibiting effects in tumors and non-cancer diseases such as inflammation, colitis, malaria, and osteoporosis (Figure 2; Figure 3; Table 1).

**TABLE 1:** Main activities of nitidine chloride and the underlying action mechanisms.

Effect	Model	Effective dose	Molecular mechanism	References
Anti-breast cancer	MDA-MB-231 cells induced in BALB/C nude mice	5 mg/kg *in vivo*	Up-modulation: p53, p21, apoptosis, Bax, cleaved caspase-9, cleaved caspase-3, cleaved PARP, E-cadherin	Pan et al. (2011); Sun et al. (2014); Sun X. X et al. (2016)
	PDGF stimulated MDA-MB-231 cells	0.1, 1, 2.5, 5, 10 μM *in vitro*	Down-modulation: MMP-9, p-c-Src, p-FAK, p-ERK, p-JNK, p-p38, RhoA, Rac1, AP-1, Bcl-2, PARP, cyclin B1, p-AKT, smo, patched 1, Gli1, Gli2, Snail, Vimentin, Slug, Zeb1, N-cadherin, Nanog, Nestin, Oct-4, CD44	
	MCF-7 cells			
	MDA-MB-231 cells			
	MDA-MB-468 cells			
Anti-ovarian cancer	A2780 cells	2.5, 5, 10, 20 μM *in vitro*	Up-modulation: apoptosis, Bax, p53, cleaved caspase-3, cleaved caspase-9, caspase-3, caspase-8, Fas, FADD	Chen et al. (2018); Ding et al. (2016); Mou et al. (2017); Sun M. J et al. (2016)
	SKOV3 cells		Down-modulation: MMP-2, MMP-9, p-ERK1/2/ERK1/2, Bcl-2, p-Akt, Skp2	
	OVCAR3 cells			
Anti-liver cancer	HCC or LC or SMMC-7721 cells xenografts in BALB/c nude mice	2.5, 5, 7, 10 mg/kg *in vivo*	Up-modulation: apoptosis, p53, p21, Bax, caspase-3	Liao et al. (2013); Liu L. M et al. (2018); Ou et al. (2015); Xiong et al. (2019)
	HepG2 cells	0.25, 0.5, 1.0 μg/ml *in vitro*	Down-modulation: proliferation, p-JAK1, p-STAT3, Bcl-2, cyclin D1, CDK4, TOP1, TOP2A, hsa_circ_0088364, hsa_circ_0090049	
	HCC cells			
	SMMC-7721 cells			
	Huh7 cells			
Anti-lung cancer	A549 cells	4, 8, 14, 32 μM *in vitro*	Up-modulation: apoptosis, arrest cell cycle, ROS, p-Mob1, p-Lats1, p-YAP	Zhang et al. (2020); Zhang et al. (2021)
	H1975 cells		Down-modulation: Mob1, Lats1, YAP, NEDD4	
	H1299 cells			
	H460 cells			
Anti-gastric cancer	SGC-7901 cells xenograft in C57BL/6 nude mice	7 mg/kg *in vivo*	Up-modulation: Apoptosis, cleaved caspase-3, PARP, miR-17-5p	Chen et al. (2012); Zhou et al. (2016)
	VEGF triggered endothelial cells	5, 10, 20, 30, 40, 60 μM *in vitro*	Down-modulation: Angiogenesis, p-VEGFR2, p-STAT3, STAT3, p-JAK1, p-JAK2, p-Src, Bcl-2, Bcl-xL, cyclin D1, VEGF, procaspase-3, CD31, c-myc	
	SGC-7901 cells			
	MKN-45 cells			
	OCUM-2MD3 cells			
Anti-renal cancer	A498 cells xenograft in BALB/c nude mice	5 mg/kg *in vivo*	Up-modulation: Apoptosis, p53, Bax, cleaved caspase-3, cleavage PARP	Fang et al. (2013); Fang et al. (2014)
	786-O cells	5, 8, 16 μM *in vitro*	Down-modulation: p-AKT, p-ERK, MMP-2, MMP-9, Bcl-2, pro-caspase-3, p-p38	
	A498 cells			
Anti-inflammation	LPS-stimulated inflammation in Raw 264.7 or THP-1 or BMDCs cells	0.5, 1, 2, 4, 5 μM *in vitro*	Up-modulation: IκBα, IL-10, p-Akt, p-GSK3β	Wang et al. (2012); Yang X. G et al. (2019)
			Down-modulation: TNF-α, IL-1β, IL-6, nucleus p65, p-ERK, p-JNK	
Anti-colitis	DSS induced colitis in C57BL/6 mice	7.27 mg/kg *in vivo*	Down-modulation: miR-31, NF-κB, COX-2	Wu et al. (2019)
Anti-malaria	*Plasmodium vinckei petteri* infected CD mice	10, 20 mg/kg *in vivo*	Down-modulation: β-haematin	Bouquet et al. (2012)
	*Chloroquine-sensitive and-resistant Plasmodium strains*	120 μM *in vitro*		
Anti-osteoporosis	RANKL-induced RAW 264.7 cells	3, 6 mg/kg *in vivo*	Down-modulation: calcitonin receptor, cathepsin K, TRAP, NFATc1, D2, NF-κB	Liu et al. (2016)
	OVX-induced bone loss in C57BL/6J mice	0.125, 0.25, 0.5, 1 μM *in vitro*		
Anti-rheumatoid	Collagen-induced arthritis in DBA/1J mice	10 mg/kg *in vivo*	Down-modulation: IL-6, IL-8, CCL-2, MMP-1, MMP-13, KCNH1, p-AKT	Shen et al. (2021)
	RA FLSs cells	0.5, 1, 2 μM *in vitro*		
Anti-psoriasis	TPA-treated BALB/c mice	1.5 μg *in vivo*	Up-modulation: p53, Bax, cleaved caspase-3, cleaved caspase-9, p-JNK, p21, apoptosis	Yang N et al. (2019)
	IMQ-treated BALB/c mice	3.25, 6.5, 13 nM *in vitro*	Down-modulation: DNA synthesis, Ki67, cyclin A, cyclin D1, Bcl-2, TNF-α, IFN-γ, IL-17A, IL-17F, IL-22, CD4, CD8, CD11b/c, CLA, CD4^+^ cells, CD8^+^ cells, CLA^+^ cells	
	HaCaT cells			
Neuroprotection	MPTP treated PD in C57BL/6 mice	2.5 mg/kg *in vivo*	Up-modulation: p-Stat3 (cytoplasm), binding activity between p-Stat3 and CRYAB, arginase 1, IL-10	Wang et al. (2016); Yuan et al. (2015)
	6-OHDA treated PD in rats	0.05, 0.1, 0.2, 0.5, 1.0 μM *in vitro*	Down-modulation: iNOS, COX-2, TNF-α, IL-1β, IL-6, MCP-1, CXCL-1, p-Jak2/Jak2, p-Stat3/Stat3, p-Stat3 (nuclear), p-ERK, p65, neuronal apoptosis	
	Stab-induced TBI model			
	Crush-induced SCI model			
	LPS induced BV2 or microglial cells			
Anti-myocardial injury	Coronary artery ligation in Wistar rats	0.5, 1, 2 mg/kg *in vivo*	Up-modulation: SOD	Wei et al. (2006)
			Down-modulation: MDA, CK, LDH, GOT, CK-MB	
Anti-renal injury	High-glucose and high-fat diet induced diabetic nephropathy in SD rats	20, 40 mg/kg *in vivo*	Up-modulation: insulin	Liu L. M et al. (2018)
			Down-modulation: UPro, UAlb, SCr, BUN, glucagon, apoptosis, p-Smad2, p-Smad3, Smad7, TGF-β1	

### Anti-cancer

#### Anti-breast cancer

In the study of Pan et al. (2011), they proved that nitidine chloride could suppress breast carcinoma cells migration and invasion. In the meantime, the protrusion formation and partial proteolytic activity of MMP-9 and MMP-2 were alleviated by nitidine chloride in a dosage-dependent mode in MDA-MB-231 cells. Moreover, administration of nitidine chloride to cells observably reduced PDGF-caused phosphorylation of c-Src, FAK, MAPKs, excitation of RhoA, Rac1 and AP-1 transcription. In conclusion, these results demonstrated that nitidine chloride possessed the potential to be a new anti-metastasis agent for breast carcinoma. In the research from Sun et al. (2014), they investigated the roles of nitidine chloride and nitidine-based combined therapy in breast carcinoma cells. The results showed that nitidine chloride caused cell growth suppression and G2/M cell cycle arrest in a time and dosage-dependent mode. Tumor cell growth suppression was related to elevated levels of the p53 and p21 proteins. Apoptosis is induced by nitidine chloride, it up-modulated the apoptosis-promoting proteins Bax, cleaved caspase-9 and -3 and cleaved PARP, and down-modulated the anti-apoptosis proteins Bcl-2 and PARP. Moreover, nitidine-caused apoptosis may be Akt-specific or dependent. Besides, nitidine chloride had a synergic action with doxorubicin on the growth suppression of breast carcinoma cells. Sun X. X et al. (2016) investigated the effect of nitidine chloride on the EMT and the CSCs-like properties in breast carcinoma cells, and the potential mechanism. The results showed that nitidine chloride could restrain the constituents of Hedgehog path (smoothened, patched, Gli1 and Gli2), and then suppressed the expression of Snail, Slug and Zeb1, which were associated with the remarkable variations of the expression of EMT relevant markers (N-cadherin, E-cadherin, and Vimentin) to reverse EMT. Moreover, nitidine chloride also suppressed the expression of CSCs relevant factors including Nanog, Nestin, Oct-4 and CD44 by Hedgehog path. Besides, TGF-β1 induced increase of EMT and CSCs properties could be reversed by nitidine chloride. In conclusion, nitidine chloride inhibited breast carcinoma EMT and CSCs-like properties by suppressing Hedgehog signal path and might be a promising anti-tumor drug for breast carcinoma.

#### Anti-ovarian cancer

In the research of Sun M. J et al. (2016), they probed the anti-metastatic role of nitidine chloride on ovary carcinoma cells and the potential mechanism. The results showed that nitidine chloride restrained the proliferation, migration and invasion of A2780 ovary carcinoma cells. Nitidine chloride decreased MMP-2 and MMP-9 in a time- and dose-dependent mode. Nitidine chloride also could down-modulate ERK phosphorylation. Moreover, through adopting an ERK depressor, the role of nitidine on the level of MMP-2/9 and suppression of cell migration and invasion was confirmed. Overall, the results indicated that nitidine suppressed the migration and invasion of ovary carcinoma cells by the ERK signal path. Ding et al. (2016) demonstrated that nitidine chloride inhibited the proliferation of ovary carcinoma cells and caused the apoptosis, and the role was mediated by the Akt signal path. Furthermore, nitidine possessed a synergic action with doxorubicin in ovary carcinoma cells. Mou et al. (2017) investigated the mechanism of nitidine-mediated tumour inhibitory action. The results proved that nitidine chloride markedly restrained the expression of Skp2 in ovary carcinoma cells, and over expression of Skp2 counteracted the anti-tumor effect caused by nitidine in ovary carcinoma cells. Strikingly, decline of Skp2 expression increased the sensitivity of ovary carcinoma cells to nitidine chloride. Therefore, devitalization of Skp2 by nitidine could be a new method for treating ovary carcinoma. Chen et al. (2018) also explored the apoptotic action and potential mechanism of nitidine in epithelial ovary carcinoma. The results indicated that nitidine restrained the proliferation of SKOV3 cells and caused apoptosis. Moreover, nitidine chloride increased the level of Fas, FADD, caspase-8 and caspase-3. After silencing caspase-8, the antiproliferative and pro-apoptotic activity of nitidine in SKOV3 cells reduced. In conclusion, nitidine induced apoptosis in SKOV3 cells through exciting the Fas signal path, and caspase-8 exerted a significant action in this process.

#### Anti-liver cancer

In the research from Liao et al. (2013), they used HCC murine xenograft model to assess the action of nitidine chloride on cancer growth and probe the possible mechanism. The results showed that nitidine chloride reduced the tumour volume and weight, manifesting that nitidine chloride suppresses HCC cell growth. Moreover, nitidine chloride blocked the excitation of JAK1-STAT3 in the tumour tissue, which in turn led to the induction of neoplasm cell apoptosis and the suppression of proliferation. Thus, nitidine chloride decreased the expression of cyclin D1, CDK4 and Bcl-2, and enhanced the expression of p21 and Bax. Ou et al. (2015) explored the specific mechanism of nitidine chloride against hepatocellular carcinoma. The results elucidated an apoptotic path in SMMC-7721 cells, associated with G_2_/M arrest, up-modulation of p53, Bax, caspase-3 and p21, and down-modulation of Bcl-2. Liu L. M et al. (2018) determined the effect of TOP1 and TOP2A in hepatic carcinoma, and investigated the inhibiting action of nitidine chloride on these two topoisomerases. The results indicated that TOP1 and TOP2A are oncogenes in hepatic carcinoma and could serve as promising markers for predicting the prognosis of hepatoma sufferers and identificating high-risk cases, thus optimizing individual therapy management. Notably, it was concluded that TOP1 and TOP2A are promising drug targets of nitidine chloride treating hepatic carcinoma. In another study, Xiong et al. (2019) explored the mechanism of nitidine chloride against hepatocellular carcinoma. The results proved that the crosstalk between hsa_circ_0088364 and hsa_circ_0090049 and their competing mRNAs may play significant roles in hepatocellular carcinoma, manifesting that circRNAs are promising therapeutical targets of nitidine chloride in hepatocellular carcinoma.

#### Anti-lung cancer

Zhang et al. (2021) studied the role and mechanism of nitidine chloride against lung cancer. The results showed that nitidine chloride could suppress cell activity, migration and invasion, but induce apoptosis in lung carcinoma cells. The mechanistic investigation manifested that nitidine chloride exerted the anticancer role through decreasing NEDD4 expression. Moreover, the rescue experiment proved that over-expression of NEDD4 counteracted the nitidine-mediated anticancerous role in lung carcinoma cells. Therefore, downmodulation of NEDD4 heightened the nitidine-caused antitumor activity. In another study, Zhang et al. (2020) explored the anti-cancer role and mechanism of nitidine chloride in NSCLC cells. The results indicated that nitidine chloride restrained the growth, motility of NSCLC cells, induced apoptosis and arrested cell cycle. In the meantime, nitidine chloride elevated the content of ROS in NSCLC cells. Furthermore, nitidine chloride inhibited the expression of Lats1, Mob1 and YAP, and increased the expression of p-Lats1, p-Mob1, p-YAP1 (ser127). These results revealed that nitidine chloride exerted the antitumor role through triggering and regulating the Hippo signal path. Collectively, nitidine chloride might be a potential anticancer agent in lung carcinoma.

#### Anti-gastric cancer

STAT3 is tightly related with malignant tumors, and constitutive activation of STAT3 exerts a critical action in cell survival, angiogenesis, immune escape and inflammation. In the study of Chen et al. (2012), they firstly proved that nitidine chloride inhibits gastric cancer growth and cancer angiogenesis through restraining constitutively activated STAT3 signal cascade. Zhou et al. (2016) also studied the mechanism of nitidine chloride against gastric cancer. The results showed that downregulation of STAT3 and c-myc and overexpression of miR-17-5p decreased side population after nitidine chloride administration. Subsequently, nitidine chloride decreased side population through inhibiting STAT3/c-myc signal *via* miR-17-5p in gastric carcinoma cells.

#### Anti-renal cancer

In the research of Fang et al. (2013), they probed the anti-metastasis effect of nitidine chloride in kidney carcinoma cells and the possible mechanism. The results proved the anti-metastasis role of nitidine chloride on kidney carcinoma cells *in vitro*. Moreover, nitidine chloride effectively restrained the migration and invasion of 786-O and A498 cells. Mechanistically, it was discovered that nitidine chloride markedly reduced phosphorylation of AKT, along with decline of MMP-2 and MMP-9. In addition, a specific AKT depressor, LY294002, could strengthen the anti-metastasis role of nitidine chloride, manifesting that nitidine chloride inhibited metastasis of kidney carcinoma cells partly by suppression of Ala activity. Fang et al. (2014) explored the anti-tumor role of nitidine chloride and the potential mechanism in kidney carcinoma cells. The growth inhibitory and pro-apoptotic roles of nitidine chloride on kidney carcinoma cells were testified both *in vitro* and *in vivo*. Furthermore, nitidine chloride effectively restrained the growth of 786-O and A498 cells in a time- and dosage-dependent mode. The xenograft model conducted in nude murines also displayed reduced cancer growth after nitidine chloride therapy. Mechanically, nitidine chloride observably reduced phosphorylation of ERK and Atk, along with up-modulation of P53, Bax, cleavage caspase-3 and cleavage PARP, down-modulation of Bcl-2, caspase-3 and PARP. Moreover, a specific MEK suppressant, PD98059, could strengthen the pro-apoptotic roles of nitidine chloride, indicating that nitidine chloride might activate apoptosis in kidney carcinoma cells partly by suppression of ERK activity. In summary, nitidine chloride could be developed as a promising antitumor drug for kidney carcinoma.

### Anti-inflammation

Wang et al. (2012) determined the anti-inflammatory activities and mechanism of nitidine chloride in murine macrophages. The results showed that nitidine chloride observably decreased the generation of pro-inflammatory cytokines such as TNF-α, IL-1β, and IL-6 in both RNA and proteic level. Furthermore, transcriptional activity of NF-κB and the phosphorylation of MAPKs in LPS-stimulated RAW 264.7 was notably restrained by nitidine chloride in a dosage-dependent mode. Besides, nitidine chloride exerts its anti-inflammatory activity through suppressing TNF-α, IL-1β and IL-6 generation and NF-κB and MAPK signal paths in RAW 264.7 cells. These results indicated that nitidine chloride restrains LPS-induced TNF-α, IL-1β and IL-6 generation by the inhibition of phosphorylation of MAPK and the translocation of p65. In conclusion, this study uncovered a new effect of nitidine chloride in modulation of inflammatory disorders.

In the study from Yang X. G et al. (2019), they discovered that nitidine chloride increased IL-10 level in LPS-treated myeloid cells. Although nitidine chloride could inhibit TOP1, nitidine chloride analogs could not suppress TOP1 failed to enhance IL-10 level. Furthermore, TOP1 depressors TPT and SN-38 also markedly increased IL-10 level, and knockdown of TOP1 stopped NC, TPT and SN-38 from elevating IL-10 expression. Therefore, nitidine chloride facilitated IL-10 generation *via* restraining TOP1. In LPS-treated endotoxemic murines, nitidine chloride and TOP1 suppressants enhanced IL-10 generation, restrained inflammatory reactions, and decreased mortality markedly. The anti-inflammatory effects of TOP1 suppression were notably decreased by IL-10-neutralizing antibody and mostly absent in IL-10 deficient murines. In LPS-treated RAW264.7 cells and in peritoneal macrophages from endotoxemic murines, nitidine chloride and TOP1 depressors prominently increased the excitation of Akt, a vital signal transducer for IL-10 generation, and suppression of Akt stopped these compounds from elevating IL-10 level and relieving endotoxemia. In conclusion, nitidine chloride and TOP1 suppressants could exert anti-inflammatory effect by elevating Akt-mediated IL-10 generation and may contribute to the therapy of inflammation disorders.

### Anti-colitis

In the research of Wu et al. (2019), they investigated the protecting action of nitidine chloride on DSS-induced ulcerative colitis in mice by targeting miR-31 and its potential mechanism. The results showed, compared with the DSS group, the DAI scores in the DSS + nitidine group were reduced, and the pathologic damage in the colons was significantly relieved after administration of nitidine chloride. Compared with normal group, the expression of miR-31 in colon tissue of the DSS group was elevated, and the expression of miR-31 was notably lowered after nitidine chloride treatment. Moreover, in comparation with the DSS group, the levels of inflammatory protein NF-κB and COX-2 in the DSS + nitidine group was prominently reduced. Collectively, nitidine chloride has remarkable therapeutical effect on DSS-treated ulcerative colitis, and the anti-inflammatory mechanism is associated with the down-modulation of miR-31 expression.

### Anti-malaria

In the study of Bouquet et al. (2012), they explored the effect and underlying mechanism of nitidine chloride against malaria. Based on the results, nitidine chloride displayed comparable effect in chloroquine-sensitive and resistant strains *in vitro*, and exhibited adequate selectivity index in comparation with a non-cancerous cell line. During therapy, there was no evidence of toxicity. The parasite cycle showed that nitidine had no effect on DNA replication, which was in line with the discovery that nitidine was localized in the cytoplasm of the parasite rather than the nucleus. *In vitro*, nitidine chloride formed a 1-1 complex with haem and restrained the formation of β-haematin (IC_50_ = 18 ± 7 μm), which exhibited superior inhibitory effect than positive control quinine (IC_50_ = 78 ± 20 μm) and a capacity comparable with chloroquine (28 ± 5 μm). Taken together, nitidine chloride was a promising anti-malaria drug, and its capacity to bind haem and suppress β-haematin formation manifests a mechanism similar to that of chloroquine.

### Anti-osteoporosis

In the research of Liu et al. (2016), they explored the role of nitidine chloride on osteoclastogenesis, bone resorption and RANKL-activated NF-κB and NFATc1 signal. In mice derived BMMs, nitidine chloride restrained RANKL-induced multinucleated TRAP-positive osteoclast formation and bone resorption in a dosage-dependent mode. Nitidine chloride alleviated the expression of osteoclast marker genes involving cathepsin K, D2, calcitonin receptor, NFATc1 and TRAP. Moreover, nitidine chloride suppressed RANKL-induced NF-κB and NFATc1 signal paths. *In vivo* research proved that nitidine chloride counteracted oestrogen deficiency-caused bone reduction in ovariectomized murines. Histologic experiment indicated that the quantity of osteoclasts was notably reduced in nitidine-treated groups. Significantly, NC at a dose of 6 mg/kg was equivalent to estrogen in protecting bone loss in OVX-induced mice bone loss. Altogether, nitidine chloride alleviated osteoclastogenesis and OVX-caused bone reduction through suppressing RANKL-activated NF-κB and NFATc1 signal paths. Nitidine chloride might be a potential agent for the therapy of osteoporosis.

### Anti-rheumatoid

In the study of Shen et al. (2021), they investigated the action of nitidine chloride in controlling FLS-mediated synovial inflammation and joint destruction in rheumatoid arthritis and further probed the potential mechanism. The results showed that nitidine chloride decreased the proliferation, migration, invasion and lamellipodia formation but not apoptosis of rheumatoid arthritis FLSs. Nitidine chloride also inhibited TNF-α induced expression and secretion of IL-6, IL-8, CCL-2, MMP-1, and MMP-13. Moreover, Shen et al. found a gene (KCNH1) that encodes ether-a-go-go-1 channel, as a new targeting gene of nitidine chloride in rheumatoid arthritis FLSs. KCNH1 expression was elevated in FLSs and synovial tissues of rheumatoid arthritis sufferers in comparation with healthy controls. KCNH1 knockdown or nitidine chloride administration reduced the TNF-α-activated AKT phosphorylation. Strikingly, nitidine chloride administration relieved the seriousness of arthritis and decreased synovial KCNH1 expression in collagen-induced arthritis mice. Overall, nitidine chloride restrained aggressive and inflammatory role of rheumatoid arthritis FLSs through targeting KCNH1 and sequential suppression of AKT phosphorylation, manifesting that nitidine chloride may control FLS-mediated rheumatoid synovial inflammation and joint destruction and be a new therapeutical drug for rheumatoid arthritis.

### Anti-psoriasis

In the study of Yang N et al. (2019), they explored the action and mechanism of nitidine chloride against psoriasis in human HaCaT keratinocytes. The results showed, nitidine suppressed HaCaT proliferation and caused S phase cell cycle arrest, which were related to the decreased productions of cyclin A, cyclin D1, and Ki67, inhibited DNA synthesis, and elevated p53 level. Moreover, nitidine observably downmodulated bcl-2 and upmodulated bax, cleaved-caspase-9, -3. Mechanism research indicated that apoptosis caused by nitidine is related to JNK path. Noticeably, nitidine restrained murine ear and dorsum skin swelling in TPA- and IMQ-treated epidermic hyperplasia and inflammatory models. Its protective effect was equivalent to that of the positive control drug dexamethasone. Besides, nitidine could relieve angiogenesis and abundant inflammatory cells infiltrated around the blood vessels. Moreover, nitidine reduced the levels of pro-inflammatory factors, including TNF-α, IFN-γ and IL-22, in the above two models. Taken together, nitidine chloride may be a potential therapeutic agent of psoriasis through suppressing HaCaT proliferation, induced apoptosis partly by JNK signal path *in vitro* and relieving skin injury and inflammation *in vivo.*

### Neuroprotection

In the study of Yuan et al. (2015), they found that nitidine chloride restrained reactive microgliosis and facilitated CNS renovation after trauma. Nitidine chloride was proved to stop cultured microglia from LPS-stimulated reactive activation through modulation of ERK and NF-κB signal path. Moreover, nitidine mediated suppression of microgliosis was also displayed in impaired brain and spinal cord, which prominently elevated neuron survival and reduced nervous tissue damage after injury. Noticeably, behavior analysis indicated that nitidine treated murines with SCI had ameliorative function restoration as evaluated by Basso Mouse Scale and swimming experiment. Collectively, these results manifested that nitidine chloride enhanced CNS tissue sparing and promoted function restoration through alleviating reactive microgliosis, suggestive of the promising therapeutical effect for CNS damage.

Wang et al. (2016) explored the effect of nitidine in neuroinflammation of PD and the underlying mechanism. In this research, nitidine chloride markedly inhibited neurotoxin-induced microglia excitation *in vitro*. Moreover, it was proved that the inhibiting role of nitidine chloride on microglia excitation is mediated by the Jak2-Stat3 path and that nitidine chloride could restrain the nuclear transsituation of p-Stat3 through elevating the binding activity of CRYAB. Strikingly, nitidine chloride could suppress reactive microgliosis and protect dopaminergic neurons in two PD animal models. Furthermore, it was indicated that nitidine chloride observably increased the neurobehavior activity in PD animal models. Overall, nitidine chloride could prominently inhibit microglial excitation to exert its neuroprotective effect on PD by the Jak2-Stat3 path.

### Anti-myocardial injury

In the study of Wei et al. (2006), they explored the effect of nitidine chloride on myocardial ischemia/reperfusion damage in rats. The results showed that 1 and 2 mg/kg nitidine chloride could decrease the occurrence rate of cardiac arrhythmia in murines with myocardial ischemia and reperfusion, postpone the emergence time of cardiac arrhythmia and shorten its duration, reduce the degree of ST segment elevation after reperfusion for fifteen and 60 min, which possess similar action with verapamil. Moreover, 1 and 2 mg/kg nitidine chloride could decrease the release of myocardial enzyme, alleviate the seriousness of oxygen-derived free radicals damage, and exerts the action of protecting myocardial damage during ischemia-reperfusion, in which represents a dosage-dependent role.

### Anti-renal injury

Liu L. M et al. (2018) studied the protective role of nitidine chloride on renal injury in diabetic nephropathy rats, and probed the possible mechanism. The results showed that the insulin level in the diabetic nephropathy group was lower than that in the control group, and the glucagon level was higher than that in the control group. After administration of nitidine chloride, the content of insulin in serum increased and the content of glucagon decreased significantly. PAS staining displayed weak positive staining and diffuse glomerular sclerosis in the low-dose group and no thickening matrix structure and renal tubular lesion was observed in the high-dose group. Moreover, Western blotting revealed that the phosphorylation of Smad2 and Smad3 and the proteic expression of TGF-β1 and Smad7 in kidney tissues were markedly reduced after nitidine chloride treatment. In summary, nitidine chloride could inhibit kidney damage in diabetic nephropathy rats by suppression of TGF-β/Smad signal path.

### Urease inhibition

In the research of Lu et al. (2021), they explored the inhibiting role, kinetics and possible mechanism of nitidine chloride against jack bean urease. The results indicated that nitidine acted as a time- and concentration-dependent depressor with an IC_50_ value of 33.2 ± 4.8 μm and exerted a similar inhibiting role to acetohydroxamic acid (IC_50_ = 31.7 ± 5.8 μm). Kinetic study suggested that nitidine was a slow binding and noncompetitive restrainer against enzyme. Thiol protectants, such as dithiothreitol, glutathione and L-cysteine, could remarkablely suppress nitidine induced urase inactivation, while Ni^2+^ competitive retardants, including boric acid and sodium fluoride, synergistically inhibited urase activity with nitidine. This result indicated that the inhibition mechanism of nitidine on urase may be closely related to its inhibition of the sulfhydryl group at the urase active site, which was further confirmed by the molecular docking technology. In addition, nitidine-induced enzyme inactivation was reactivated by glutathione, indicating that the inhibition of nitidine on urase was reversible. In summary, nitidine is a promising new urease suppressant with the characteristics of reversibility and slow binding, and noncompetitive depressor targeting the sulfhydryl group in the urase active site.

## Pharmacokinetics of nitidine chloride

In the study from Chen et al., they determined the equilibrium solubility of nitidine chloride in different solvents and surfactant solutions and the apparent oil-water partition coefficient in n-octanol-water/buffer liquid system (Chen et al., 2010). The equilibrium solubility of nitidine chloride in water, methanol, absolute ethanol, 60% ethanol and petroleum ether at 37 °C is 363.72, 1047.23, 301.92, 695.62, and 2.89 mg L^−1^, respectively. Poloxamer 188, Tween-80 and PEG-400 have strong solubilization ability to nitidine chloride, and the apparent oil-water distribution coefficient of nitidine chloride was 54.61. These results indicated that the water solubility of nitidine chloride is poor, and surfactant can improve the solubility of nitidine chloride in water. Zhao et al. (2018) evaluated the absorption of nitidine chloride in the small intestine of rats. The results manifested that the absorption of nitidine chloride in rat small intestine is independent of the drug concentration, and the intestinal absorption of nitidine chloride *in vivo* is passive diffusion.

Ye et al. (2008) established a HPLC method for the determination of nitidine chloride in human plasma, and investigated the binding of nitidine chloride to human plasma proteins. The results showed that nitidine chloride has a moderate protein binding rate, which is independent of the drug concentration in dialysate. Moreover, in the studies of rat and rabbit plasma, they also got similar conclusions (Ye et al., 2009b; Ye et al., 2010). In another research, Ye et al. (2009a) established a HPLC method to determine nitidine chloride in rat urine and feces, and studied its excretion process in rats. The results indicated that nitidine chloride is rarely excreted with urine and feces in the form of prototype drug.

Liu et al. (2009b) established a HPLC method for the determination of nitidine chloride in rat plasma and tissues to study its tissular distribution. The results showed that the drug was rapidly and widely distributed in rats after intravenous injection, and distributed in various tissues and organs within 5 min. The concentration of drugs in kidney was the highest, followed by small intestine and liver. In another study, Liu et al. (2009a) established a HPLC method for the determination of nitidine chloride in rabbit plasma and investigated the pharmacokinetics of nitidine chloride by intravenous injection in rabbits. The results showed, after intravenous injection of nitidine chloride in rabbits, the drug-time curve accorded with the two-compartment model. In 4, 6 mg kg^−1^ dose groups, T_1/2α_ is severally (5.46 ± 0.89) (4.76 ± 0.33) min, T_1/2β_ is severally (263.33 ± 16.34) (274.71 ± 16.52) min, AUC is severally (46.56 ± 1.80) (69.19 ± 2.30) μg·min^−1^·mL^−1^. These results manifested that the elimination of nitidine chloride was linear in this dose range.

Wang et al. (2017) investigated the pharmacokinetics of nitidine chloride in rat plasma after intragastrical administration. The results showed that a LC-ESI-MS/MS method was effectively employed to the pharmacokinetic research after a single dosage of nitidine chloride at 25 mg/kg by intragastrical administration to murines. The parameters from the dosage were discovered to fit best a two-compartment open model. The plasma concentration maximum (*C*_max_) of nitidine chloride was (96.48 ± 5.30) ng/ml and separately reduced to (41.16 ± 3.19) ng/ml and (10.46 ± 1.01) ng/ml at 4 and 8 h post-dosing, and the time to *C*_max_ was 2 h (*T*_max_), manifesting that the absorption of nitidine chloride was slower with moderate elimination.

## Toxicology of nitidine chloride

**FIGURE 4:**
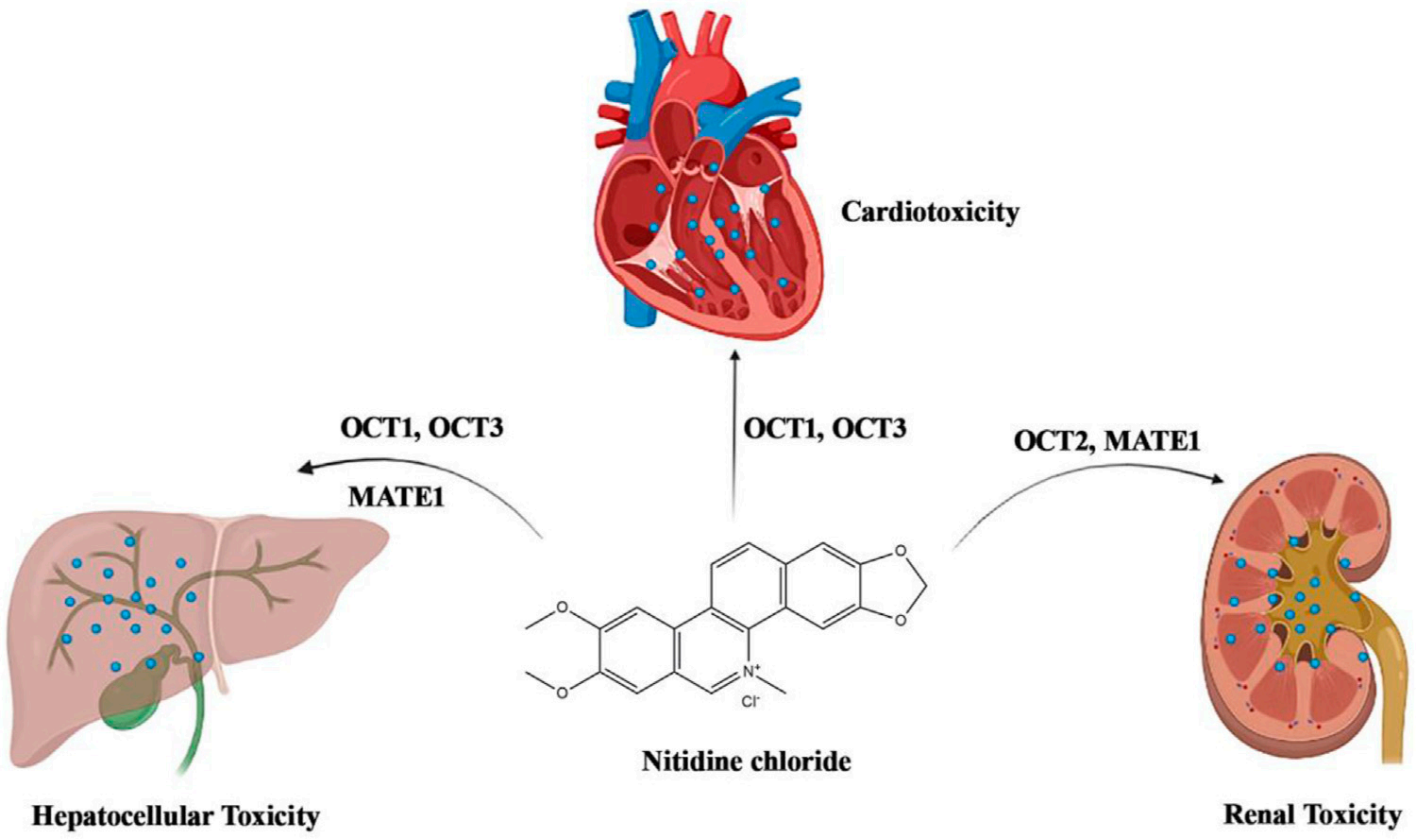
Toxic mechanisms of nitidine chloride on kidney, heart, and liver.

Although nitidine chloride shows a wide range of pharmacological activities, especially in the anti-cancer effect, some studies have indicated that nitidine chloride has certain toxicity to the heart, liver and kidney (Figure 4).

### Renal toxicity

Wei et al. (2009) used MTT method to detect the toxicity of nitidine chloride at different concentrations on human embryonic kidney cell 293. The results showed that nitidine chloride could inhibit the proliferation of human embryonic kidney cell 293 and had certain toxicity to the kidney. In the study of Li et al. (2016), they evaluated the action of OCT2 and MATE1 in the nephric disposition and nephrotoxicity of nitidine chloride. The results showed that nitidine chloride is a high-affinity substrate of both OCT2 and MATE1 with high cytotoxicity in MDCK-hOCT2/hMATE1 and MDCK-hOCT2 in comparation with mock cells. The OCT2 depressors, cimetidine and (+)-THP, markedly decreased nitidine chloride accumulation and cytotoxicity in MDCK-hOCT2, MDCK-hOCT2/hMATE1 and rPCPT cells. Serious renal injury with high contents of BUN and LDH, decreased levels of ALP and pathologic variations were discovered in murines after nitidine chloride treatment. In conclusion, nitidine-induced nephrotoxicity was tightly associated with OCT2-modulated extensive nephric absorption and feeble tubular secretion by MATE1.

### Cardiac toxicity

Huang X. Y et al. (2011) observed the effects of nitidine chloride at different concentrations on zebrafish embryo hearts. At 60 h and 72 h after fertilization, fetal cardiac toxicity was observed in all treatment groups. In the 5.00 mg/L and 3.15 mg/L experimental groups, the embryos mainly showed cardiac arrest and bleeding in the heart area, while the embryos in the 2.00 mg/L, 1.58 mg/L and 1.12 mg/L experimental groups mainly showed cardiac developmental malformation. The heart rate decreased significantly with the increase of concentration, but not significantly with the extension of exposure time. Therefore, nitidine chloride is cardiotoxic to zebrafish embryos. In the research of Li et al. (2019), they investigated the distribution of nitidine chloride in rat heart and the possible cardiotoxicity. The results showed that nitidine chloride accumulates in rat heart and has certain toxicity to primary cardiomyocytes and fibroblasts, and OCT1 and OCT3 can regulate the cardiac accumulation of nitidine chloride.

### Hepatic toxicity

In the study of Li et al. (2014), they evaluated the effect of OCT1, OCT3, MATE1, and P450 enzymes on nitidine-caused hepatotoxicity. The datas indicated that the absorption of nitidine chloride in MDCK-hOCT1/MDCK-hOCT3 was notably better than that in mock cells, and the hOCT1 and hOCT3 mediated absorption fitted Michaelis-Menten dynamics. Simultaneously, nitidine chloride was also a substrate of hMATE1, though its transport ability was extremely less than that of OCT1. Nitidine caused much higher cytotoxity in MDCK-hOCT1 or MDCK-hOCT3 cells than in mock cells. Quinidine and depressors of (+)-THP, OCT1 and OCT3, observably decreased the absorption of nitidine in MDCK-hOCT1/MDCK-hOCT3 cells and murine primary hepatic cells, while only (+)-THP notably alleviated the nitidine-caused virulence. Moreover, P450 enzymes (e.g. CYP3A4) could regulate the metabolism of nitidine, and nitidine-caused virulence in MDCK-hOCT1/hCYP3A4 cells was less than that in MDCK-hOCT1 cells. Collectively, nitidine chloride is a substrate of OCT1, OCT3 and CYP3A4, which regulate the absorption of nitidine in hepatic cells and then induce liver toxicity. Moreover, nitidine-caused toxicity can be relieved by CYP3A4 modulated metabolism.

## Formulation of nitidine chloride

Solid lipid nanoparicles (SLN) are colloidal drug delivery systems with particle size of about 50–1000 nm, which are made of natural or synthetic solid lipids as carriers. They have the merits of high physical stability, sustained release and good targeting, and are a promising drug delivery system. In order to give full play to the antitumor activity of nitidine chloride and improve drug targeting, Chen et al. (2011) successfully prepared nitidine-SLN, and compared the pharmacokinetics of nitidine-SLN and nitidine chloride in rats. The results showed, compared with free nitidine chloride, nitidine-SLN have the characteristics of long-term and slow-release.

Ye et al. (2019) explored the best technological conditions for the preparation of nitidine chloride-phospholipid complex. The results showed that the optimum preparation conditions were as follows: methanol was used as solvent, the concentration of nitidine chloride was 0.5 mg/ml, the feed ratio of nitidine chloride to phospholipids was 1:3 (mol/mol), the reactive temperature was 40°C, and the reactive time was 1.5 h. This condition can make the binding rate of nitidine chloride and phospholipids reach more than 85%. The results showed that the preparation process of the complex was stable and feasible, and the binding rate was high, which provided a reference for the pharmaceutical research of nitidine chloride.

In order to reduce toxicity and increase efficiency by embedding nitidine chloride in microspheres, Zhang (2019) conducted investigation of the preparation technology, content determination and quality research of nitidine chloride microspheres. On this basis, the quality standard of nitidine chloride microspheres was formulated. The results indicated that the PLGA microspheres of nitidine chloride prepared by optimizing the formulation have certain drug loading, high entrapment efficiency and obvious *in vitro* sustained-release effect, providing reference and guidance for the later development and clinical application of the preparation.

As is well-known, nitidine chloride possesses excellent anticancer effect. Whereas, its clinic practice is restricted due to the aspecific toxicity and poor bioavailability. In the research of Bi et al. (2020), they prepared nanoscale metal-organic frameworks MIL-100(Fe), which was adopted as a nanocarrier to deliver nitidine chloride. The results showed that MIL-100(Fe) had a high drug capacity (33.43 wt%) for nitidine chloride by the impregnation method. The release of nitidine chloride in PBS manifested progressive drug release, with 68% released within 4 days. Moreover, nitidine@MIL-100(Fe) displayed higher suppression than nitidine chloride in HEPG2 cells, with lower toxicity found in the normal liver cell line LO2. Therefore, MIL-100(Fe) is a promising drug delivery carrier that can relieve the systemic adverse reactions of nitidine chloride and increase the antitumor effect *in vitro*. In conclusion, the nitidine@MIL-100(Fe) was effective in nitidine chloride delivery and can help move nitidine chloride further through the drug development pipeline.

Li et al. (2021) investigated TPGS-FA as a promising carrier for controlled delivery of nitidine chloride. TPGS-FA/nitidine complexes were made smoothly, and were homogenious with a consistent size of 14 nm diameter. Nitidine chloride was discharged from the TPGS-FA/nitidine complexes in a controlled and sustained mode under physiologic conditions. Moreover, its inhibitory action to hepatoma cells was higher than that of free nitidine chloride. These results indicated that TPGS-FA is an excellent carrier for nitidine chloride, and TPGS-FA/nitidine might be an effective and safe preparation to treat liver cancer.

## Conclusion

Nitidine chloride is a benzophenanthridine alkaloid existed in medicinal plants, and mainly extracted from the roots or stems of *Z. nitidum*, which is a famous traditional Chinese medicine usually used for the treatment of gastrointestinal diseases, toothache, and traumatic injury. Enzymatic and ultrasonic methods are recommended for the extraction of nitidine chloride, resin adsorption and chromatography were usually used for the separation and purification of nitidine chloride. Nitidine chloride possesses diversified therapeutical effects, such as anti-cancer, anti-inflammation, anti-colitis, anti-malaria, anti-osteoporosis, anti-rheumatoid and so on. However, nitidine chloride has a few limitations including poor solubility, low bioavailability and certain toxicity, which could be effectively resolved through new formulations, such as nanoparticle, microsphere and nano-micelle in addition to studies of ADME/Tox properties. Therefore, nitidine chloride has great potential to be developed as a new drug, especially in anti-cancer.

## Glossary

ALP: alkaline phosphatase
Bax: Bcl-2-associated X protein
Bcl-2: B-cell CLL/lymphoma 2
BMMs: bone marrow monocytes
BUN: blood urea nitrogen
CDK: cyclin-dependent kinase
CK: creatine kinase
CK-MB: creatine phosphokinase isoenzyme
CNS: central nervous system
CRYAB: αB-crystalline
CSCs: cancer stem cells
DSS: dextran sodium sulfate
EMT: epithelial mesenchymal transition
ERK: extracellular signal-regulated kinase
FADD: Fas-associated death domaincontaining protein
FLS: fibroblast-like synoviocytes
GOT: glutamic-oxalacetic transaminase
GSK3β: glycogen synthase kinase 3β
HCC: hepatocellular carcinoma
IMQ: imiquimod
JAK: Janus-activated kinase
JNK: c-Jun N-terminal kinase
Lats: large tumor suppressor kinase
LDH: lactate dehydrogenase
LPS: lipopolysaccharide
MAPKs: mitogen-activated protein kinases
MATE1: multidrug and toxin extrusion 1
MMP: matrix metalloproteinase
Mob1: Mps one binder protein 1
NEDD4: neuronally expressed developmentally downregulated 4
NSCLC: non-small cell lung cancer
OCT2: organic cation transporter 2
OVX: ovariectomy
PAS: periodic acid Schiff
PCNA: proliferating cell nuclear antigen
PD: Parkinson’s disease
ROS: reactive oxygen species
SCI: spinal cord injury
Skp2: S-phase kinase associated protein 2
Sm: smoothened
STAT3: Signal transducer and activator of transcription 3
TBI: traumatic brain injury
TGF-β1: transforming growth factor-β1
THP: tetrahydropalmatine
TOP1: topoisomerase I
TOP2A: topoisomerase 2A
TPA: 12-O-tetradecanoyl-phorbol-13-acetate
TPGS-FA: folic acid modified D-α-tocopheryl polyethylene glycol 1000 succinate
TRAP: tartrate-resistant acid phosphatase
YAP: Yes-associated protein
